# Lymph node metastasis in cutaneous squamous cell carcinoma of the head and neck

**DOI:** 10.1186/s12885-024-12384-6

**Published:** 2024-05-29

**Authors:** Qigen Fang, Junhui Yuan, Xu Zhang, Liyuan Dai, Ruihua Luo

**Affiliations:** 1 Department of Head Neck and Thyroid, The Affiliated Cancer Hospital of Zhengzhou University & Henan Cancer Hospital, Zhengzhou, 450008 China; 2 Department of Radiology, The Affiliated Cancer Hospital of Zhengzhou University & Henan Cancer Hospital, Zhengzhou, 450008 China

**Keywords:** Intraparotid lymph node, Periparotid lymph node, AJCC, O'Brien stage, Cutaneous squamous cell carcinoma

## Abstract

**Background:**

The study aimed to assess the impact of parotid lymph nodes (LNs) on the prognosis of patients with cutaneous squamous cell carcinomas of the head and neck (HNcSCC), and to develop an alternative LN assessment method to enhance locoregional control (LRC) and overall survival (OS) stratification.

**Methods:**

We retrospectively enrolled patients with surgically treated HNcSCC. Primary outcome variables were LRC and OS. The influence of parotid LNs and different LN assessment methods on prognosis was analyzed using Cox models, and comparisons were made using the C-index, Akaike Information Criterion, and Bayesian Information Criterion.

**Results:**

A total of 126 patients were included. Both intraparotid and periparotid LN statuses significantly linked with prognosis. The presence of extranodal extension (ENE) in cervical LNs, rather than parotid LNs, was predictive of decreased LRC and OS. In the Cox analysis, only N3 of the AJCC N classification, when compared to N0, showed reduced LRC and OS. In comparison to N0P1, only N0P3/N1P1 and N2P2/N2P3 of the O’Brien staging system tended to predict poorer LRC, with no subgroup emerging as an independent predictor for OS. The proposed LN assessment method, based on the number of metastatic LNs and ENE status in cervical LNs, demonstrated superior performance in terms of C-index, Akaike Information Criterion, and Bayesian Information Criterion compared to other systems.

**Conclusion:**

Parotid LNs were significant determinants of prognosis in metastatic HNcSCC. The novel LN assessment method proposed (1–2 vs. 3–4 vs. 5 + or ENE) displayed similar survival stratification to the AJCC N and O’Brien staging systems.

## Background

Cutaneous squamous cell carcinoma is a prevalent form of skin cancer, with a significant portion arising in the head and neck region (HNcSCC), often links to factors such as sun exposure and immunosuppression [1]. Typically, HNcSCCs have a favorable prognosis, as the lymph node (LN) metastasis rate is generally below 5%. However, in cases where the tumor size exceeds 2 cm, the depth of invasion is greater than 5 mm, or there are histological features indicating poor differentiation, additional lymphadenectomy surgery may be recommended to ensure regional control [2, 3].

The parotid LN serves as the initial drainage site for the forehead, temple, and ear regions, all of which pose a heightened risk for HNcSCC occurrence. This node complex encompasses the intraparotid and periparotid LNs. Metastasis to the parotid LN represents a significant prognostic determinant for HNcSCC [4, 5]. While the 8th edition of the AJCC classification takes into account factors such as size, number, laterality, and extranodal extension (ENE) of metastatic LNs [6], it regrettably overlooks the implications of parotid LN involvement, a lacuna that we aim to address in the following discourse.

Three distinct staging systems for parotid LN metastasis have been put forth as alternatives. The initial proposal takes into consideration the size, number, and ENE of metastases in the parotid LNs, alongside the size and number of cervical LN metastases [5]. However, the intricacy arises from the anatomical separation of parotid and cervical LNs, hindering a comprehensive stratification of all subgroups when applied to pathological data. In contrast, the N1S3 staging system, as pioneered by Forest et al. [7], factors in the size and number of metastatic LNs but regrettably overlooks ENE, a crucial consideration given its pronounced impact compared to LN size [7]. The third proposed alternative focuses solely on the count of metastatic LNs (1–2 vs. 3–4 vs. 5+), yet failed to detect a notable disparity in overall survival (OS) between subgroups with 1–2 positive LNs and those with 3–4 positive nodes [8]. Significantly, the originators of this method did not provide clarity on whether periparotid LNs were included in the resection nor did they explore the implications of periparotid LN metastasis on prognosis.

Hence, our objective was to assess the impact of parotid LN metastasis on the survival outcomes in HNcSCC, with a view to devising a novel approach for evaluating LN metastasis that yields superior delineation of locoregional control (LRC) and OS probabilities.

## Methods

### Ethical considerations

This study was approved by Our Hospital Institutional Research Committee, and written informed consent for medical research was obtained from all patients prior to initial treatment. All methods were performed in accordance with relevant guidelines and regulations.

### Study design

This study constituted a retrospective analysis of prospectively gathered data. Over the period spanning from January 2010 to December 2022, a total of 786 individuals diagnosed with primary HNcSCC and devoid of any antecedent history of malignancies underwent surgical intervention at our esteemed cancer institute. Among this cohort, 126 patients necessitated parotidectomy due to either clinically or pathologically confirmed involvement of the parotid LNs, with 57 of these individuals additionally undergoing neck dissection. Comprehensive demographic, pathological, treatment, and follow-up information was meticulously documented for all subjects.

### Study variables

Each pathological specimen underwent rigorous evaluation by a minimum of two head and neck pathologists. Tumor and nodal staging were ascertained in alignment with the 8th edition of the AJCC system. The presence of lymphovascular invasion (LVI) was affirmed if tumor cells were detected within the lymphatic vessels. Perineural invasion (PNI) was confirmed if tumor cells infiltrated the nerve tissue. ENE was diagnosed upon the identification of cancer cells beyond the confines of the lymph node capsule.

The primary outcome variables were LRC and OS. The time to LRC was calculated from the date of surgery to the date of first locoregional recurrence or last follow-up, and the time to OS was calculated from the date of surgery to the date of death or last follow-up.

### Treatment

At our distinguished oncology institution, every individual diagnosed with HNcSCC underwent ultrasound and CT examinations of both the parotid gland and the neck region. In cases where clinically or pathologically confirmed LN involvement was identified, parotidectomy and/or neck dissection were promptly carried out. During these procedures, the preauricular and infra-auricular LNs were excised as a collective unit, referred to as the periparotid lymph node, and sent for comprehensive pathological scrutiny. Adjuvant radiotherapy was prescribed in instances where LN metastasis, PNI, LVI, or other unfavorable indicators were detected. Consideration for adjuvant chemotherapy was extended to patients with positive margins or evidence of ENE.

### Statistical analysis

The relationships between clinicopathological variables and LRC and OS were initially examined through univariate Cox analysis. Subsequently, variables demonstrating significance were subjected to the multivariable Cox model. Prognostic frameworks were delineated based on diverse LN assessment approaches: the AJCC N staging, the O’Brien staging, and an optimal threshold for nodal metastases enumerated through exploratory analysis. The effects of independent variables on survival outcomes were explicated in terms of hazard ratios (HRs) accompanied by 95% confidence intervals (CIs). The reliability and discriminatory prowess of the prognostic models were assessed utilizing the Akaike Information Criterion (AIC), Bayesian Information Criterion (BIC), and concordance index (C-index). Survival probabilities contingent on distinct LN assessment methodologies were contrasted employing the Kaplan-Meier method. Should the follow-up period exceed 5 years devoid of any outcome events, data truncation ensued at the 5-year mark. All statistical computations were executed using R 3.4.4, with statistical significance established at a threshold of *p* < 0.05.

## Results

### Baseline data

In our cohort of 126 patients, there were 100 (79.4%) male and 26 (20.6%) female participants, with a mean age of 65 ± 10 years. The primary tumor sites were distributed as follows: ear or around in 39 (31.0%) patients, temple in 35 (27.8%), forehead in 32 (25.4%), and other locations in 20 (15.9%) patients. Out of the total, 12 (9.5%) patients suffered from immunosuppression due to prior organ transplantation. Tumor staging revealed T1 in 10 (7.9%) patients, T2 in 76 (60.3%), T3 in 35 (27.8%), and T4 in 5 (4.0%) patients. PNI and LVI were identified in 36 (28.6%) and 32 (25.4%) patients, respectively. Regarding histological differentiation grade, 30 (23.8%) patients were classified as well-differentiated, 56 (44.4%) as intermediate, and 40 (31.7%) as poorly differentiated. Positive surgical margins were observed in five (4.0%) patients.

Superficial and radical parotidectomies were performed on 47 (37.3%) and 79 (62.7%) patients, respectively. Intraparotid LN metastasis was detected in 80 (63.5%) patients, among whom 20 exhibited ENE and 30 (23.8%) had positive periparotid LNs. Notably, 10 patients from the latter category also presented with ENE. The average number of metastatic parotid LNs per patient was calculated at 2 ± 2, with a median value of 2 (ranging from 1 to 6). A total of 57 patients underwent neck dissection, with 27 undergoing resection of levels I-III/IV, while the remainder received excision extending to levels I-V. Each of the 10 neck dissection specimens obtained from the 46 patients devoid of parotid metastasis displayed the presence of at least one positive LN.

The pathologic staging of the neck was delineated as follows: N0 in 71 patients (56.3%), N1 in 25 patients (19.8%), N2 in 15 patients (11.9%), and N3 in 15 patients (11.9%). Among those individuals with metastatic cervical LNs, the average number of positive nodes was determined to be 2 ± 2, with a median count of 2 (spanning from 1 to 15). A cohort of 19 patients manifested ENE.

In accordance with the O’Brien staging system, among the 71 patients (56.3%) diagnosed with pathologic N0-stage disease, instances of P1, P2, and P3 were observed in 50, 13, and 8 patients, respectively. Within the cohort of 25 patients (19.8%) with pathologic N1-stage disease, the distribution of P1, P2, and P3 were 6, 12, and 7 individuals, respectively. Moving on to the group of 30 patients (23.8%) with pathologic N2-stage malignancies, the allocation of P1, P2, and P3 were 5, 18, and 7 patients, respectively. Furthermore, utilizing the N1S3 staging system, malignancies were classified into stages I, II, and III, with 20, 73, and 33 patients falling under these respective categories.

Subsequent to a median follow-up duration of 3.6 (interquartile range: 2.0-5.3) years, adjuvant radiotherapy was administered to 100 patients, with 35 individuals additionally undergoing adjuvant chemotherapy. Among this cohort, 26 (20.8%) cases of locoregional recurrence surfaced, all confined to neck regions that had undergone prior surgical intervention. Throughout the study timeframe, a total of 22 patients succumbed.

### Univariate analysis

Given that each patient exhibited at least one metastatic LN, the entirety of the patient population was encompassed within the survival analysis. Noteworthy associations were uncovered between immunosuppression, T3/4 stage, ENE of cervical LNs, metastasis within intraparotid and periparotid LNs, neck stage, O’Brien stage, presence of positive margins, and receipt of radiotherapy, all of which demonstrated significant correlations with both LRC and OS. Age exceeding 65 years was linked to a poorer OS outcome (*p* = 0.035) as opposed to LRC (*p* = 0.105). None of other factors under investigation exhibited statistically significant associations with either LRC or OS (Table 1).

**Table 1:** Univariate Cox analysis of predictors for locoregional control (LRC), and overall survival (OS).

Variable	LRC		OS	
	*p*	HR [95%CI]	*p*	HR [95%CI]
Age				
≤65		ref		ref
> 65	0.105	1.95 [0.87–4.38]	0.035	2.75 [1.07–7.02]
Sex (Male/female)				
Female		ref		ref
Male	0.325	1.76 [0.67–4.90]	0.216	1.54 [0.75–3.86]
Primary site				
Ear or around		ref		ref
Temple	0.367	1.08 [0.54–4.35]	0.434	1.23 [0.41−5.00]
Forehead	0.278	0.96 [0.33–2.04]	0.327	0.96 [0.47–2.83]
Others	0.665	0.90 [0.31–4.38]	0.832	0.88 [0.20–6.09]
Immunosuppression				
No		ref		ref
Yes	< 0.001	9.36 [4.12–21.29]	< 0.001	11.55 [4.87–27.40]
Differentiated grade				
Well		ref		ref
Intermediate	0.839	0.89 [0.29–2.72]	0.832	0.89 [0.29–2.71]
Poor	0.144	2.16 [0.77–6.06]	0.481	1.48 [0.50–4.43]
Tumor stage				
T1/2		ref		ref
T3/4	0.001	3.99 [1.74–9.20]	0.012	3.06 [1.28–7.31]
Perineural invasion				
No		ref		ref
Yes	0.437	2.19 [0.74–7.33]	0.562	2.53 [0.63–8.14]
Lymphovascular invasion				
No		ref		ref
Yes	0.216	1.87 [0.56–5.28]	0.333	1.99 [0.45–7.18]
ENE of periparotid LN*				
No		ref		ref
Yes	0.778	1.80 [0.67–4.22]	0.892	2.00 [0.56–8.09]
ENE of intraparotid LN				
No		ref		ref
Yes	0.437	1.54 [0.71–4.86]	0.556	2.22 [0.58–8.77]
ENE of cervical LN				
No		ref		ref
Yes	< 0.001	8.69 [3.98–18.95]	< 0.001	8.22 [3.53–19.18]
Intraparotid LN metastasis				
No		ref		ref
Yes	0.018	3.61 [1.24–10.49]	0.012	6.48 [1.51–27.75]
Periparotid LN metastasis				
No		ref		ref
Yes	< 0.001	6.26 [2.86–13.68]	< 0.001	6.43 [2.74–15.12]
LN size (/)				
∼3 cm		ref		ref
3 + cm	0.756	1.88 [0.62–5.24]	0.658	1.95 [0.70–4.98]
Neck stage				
N0		ref		ref
N1	0.029	3.21 [1.12–9.17]	0.021	3.61 [1.21–10.75]
N2	0.005	4.73 [1.59–14.12]	0.049	3.53 [1.01–12.68]
N3	0.002	5.77 [1.94–17.21]	0.005	5.47 [1.67–17.97]
O’Brien stage				
N0P1		ref		ref
N0P2	0.043	6.36 [1.06–32.08]	0.157	4.13 [0.58–29.32]
N0P3/N1P1	0.002	12.33 [2.48–31.43]	0.015	8.21 [1.49–40.11]
N1P2	0.110	4.30 [0.72–25.73]	0.292	2.87 [0.40−20.37]
N1P3/N2P1	0.001	14.32 [2.88–41.05]	0.001	13.79 [2.78–38.46]
N2P2/N2P3	0.014	7.52 [1.52–27.28]	0.015	7.25 [1.46–25.98]
N1S3				
I		ref		ref
II	0.588	1.41 [0.41–4.91]	0.789	1.19 [0.34–4.21]
III	0.268	2.09 [0.57–7.73]	0.488	1.61 [0.42–6.25]
Extent of parotidectomy				
Superficial		ref		ref
Total	0.857	1.23 [0.37–6.22]	0.903	1.45 [0.41–9.88]
Positive margin				
No		ref		ref
Yes	< 0.001	24.78 [8.67–70.86]	< 0.001	26.27 [9.08–75.99]
Radiotherapy				
No		ref		ref
Yes	0.022	0.88 [0.58–0.95]	0.031	0.90 [0.60–0.99]
Chemotherapy				
No		ref		ref
Yes	0.216	1.32 [0.73–4.22]	0.532	1.67 [0.55–8.24]

* LN: lymph node; ENE: extranodal extension

Upon further investigation into LRC, the covariates of immunosuppression, tumor stage, ENE of cervical LNs, presence of positive margins, and receipt of radiotherapy were integrated into the Cox model for the AJCC N stage, O’Brien stage, and the novel proposed stage. The variables pertaining to metastasis in intraparotid and periparotid LNs were specifically assessed within the Cox model for the AJCC N stage.

Subsequently, in the extended analysis of OS, factors such as age, immunosuppression, tumor stage, ENE of cervical LNs, positive margin status, and radiotherapy treatment were included in the Cox model across the AJCC N stage, O’Brien stage, and the innovative proposed stage. The factors associated with metastasis in intraparotid and periparotid LNs were also scrutinized within the framework of the Cox model for the AJCC N stage.

### Cox model analysis

The evaluation of LNs was conducted utilizing two distinct approaches: the AJCC N staging and the O’Brien staging methods. In our investigation of LRC employing the AJCC N staging system, the presence of intraparotid LN metastasis did not serve as a predictor for worsened LRC outcomes (*p* = 0.117, HR: 2.55, 95% CI: 0.79–8.19). Conversely, periparotid LN metastasis was significantly linked to a reduction in LRC rates (*p* < 0.001, HR: 5.42, 95% CI: 2.25–13.07). Compared to N0, N1 exhibited a similar prognostic impact (*p* = 0.331, HR: 1.74, 95% CI: 0.57–5.34), while N2 showed a tendency towards diminished survival rates (*p* = 0.062, HR: 3.21, 95% CI: 0.94–10.92), and N3 significantly decreased LRC rates (*p* = 0.007, HR: 3.18, 95% CI: 1.85–11.98). Additional significant independent factors included immunosuppression, T3/4 stage, and positive margins (Table 2). The C-index was 0.645 (95% CI: 0.632–0.657). The AIC and BIC were 447 and 479, respectively.

**Table 2:** Cox models for locoregional control (LRC) based on different lymph node (N) assessment methods

LN assessment	*p*	HR [95%CI]
**Cox model for AJCC N**		
Immunosuppression	0.001	5.81 [1.96–17.17]
Tumor stage (T3−4/T1−2)	0.015	2.99 [1.24–7.22]
Intraparotid LN metastasis	0.117	2.55 [0.79–8.19]
Periparotid LN metastasis	< 0.001	5.42 [2.25–13.07]
Neck stage		
N0		
N1	0.331	1.74 [0.57–5.34]
N2	0.062	3.21 [0.94–10.92]
N3	0.007	3.18 [1.85–11.98]
Positive margin	< 0.001	14.39 [4.02–51.54]
Radiotherapy	0.111	0.89 [0.67–2.17]
**Cox model for O’Brien stage**		
Immunosuppression	0.086	3.53 [0.80−11.09]
Tumor stage (T3−4/T1−2)	0.038	2.60 [1.05–6.44]
Positive margin	< 0.001	18.93 [5.00−71.60]
ENE of cervical LN*	0.023	3.39 [1.19–9.67]
O’Brien stage		
N0P1		ref
N0P2	0.190	3.53 [0.54–23.20]
N0P3/N1P1	0.067	4.97 [0.89–27.71]
N1P2	0.290	2.72 [0.43–17.31]
N1P3/N2P1	0.397	2.16 [0.36–12.84]
N2P2/N2P3	0.059	4.96 [0.94–26.24]
Radiotherapy	0.276	0.96 [0.80–3.75]

* ENE: extranodal extension

In our analysis of LRC using O’Brien staging, in comparison to N0P1, the risk of LRC was not significantly altered by N0P2, N1P2, or N1P3/N2P1. However, N0P3/N1P1 and N2P2/N2P3 demonstrated HRs of 4.97 [95% CI: 0.89–27.71] (*p* = 0.067) and 4.96 [95% CI: 0.94–26.24] (*p* = 0.059), respectively. Other independent factors associated with LRC included immunosuppression, T3/4 stage, positive margins, and ENE of the cervical LN (Table 2). The C-index was 0.615 (95% CI: 0.604–0.626), with corresponding AIC and BIC values of 481 and 528, respectively.

Upon scrutinizing OS utilizing the AJCC N staging system, intraparotid LN metastasis was correlated with diminished survival rates (*p* = 0.045, HR: 4.64, 95% CI: 1.07–22.25), whilst periparotid LN metastasis was markedly linked to poorer OS outcomes (*p* = 0.001, HR: 5.26, 95% CI: 1.96–14.13). Among the four neck stages, N3 exhibited the highest HR of 3.83 (95% CI: 1.47-9.00), while other three stages displayed comparable HRs. Noteworthy independent variables encompassed immunosuppression, T3/4 stage, and positive margins (Table 3). The C-index was 0.633 (95% CI: 0.620–0.646). The AIC and BIC were 465 and 492, respectively.

**Table 3:** Cox models for overall survival based on different lymph node (N) assessment methods

LN assessment	*p*	HR [95%CI]
**Cox model for AJCC N**		
Age (> 65/≤65)	0.124	2.41 [0.79–7.40]
Immunosuppression	0.002	6.16 [1.92–19.76]
Tumor stage (T3−4/T1−2)	0.019	1.99 [1.45–5.06]
Intraparotid LN metastasis	0.045	4.64 [1.07–22.25]
Periparotid LN metastasis	0.001	5.26 [1.96–14.13]
Neck stage		
N0		
N1	0.406	1.67 [0.50–5.63]
N2	0.086	2.07 [0.83–17.78]
N3	0.034	3.83 [1.47−9.00]
Positive margin	< 0.001	11.79 [3.19–43.60]
Radiotherapy	0.208	0.89 [0.56–5.22]
**Cox model for O’Brien stage**		
Age (> 65/≤65)	0.042	3.16 [1.04–9.58]
Immunosuppression	0.003	5.91 [1.86–18.79]
Tumor stage (T3−4/T1−2)	0.024	2.77 [1.16–4.70]
Positive margin	< 0.001	15.69 [3.79–64.89]
ENE of cervical LN*	0.038	3.28 [1.07–10.08]
O’Brien stage		
N0P1		ref
N0P2	0.746	1.44 [0.16–12.95]
N0P3/N1P1	0.263	3.00 [0.44–20.47]
N1P2	0.618	1.67 [0.22–12.46]
N1P3/N2P1	0.449	2.03 [0.32–12.68]
N2P2/N2P3	0.090	4.29 [0.80−23.06]
Radiotherapy	0.246	0.92 [0.54–5.05]

* ENE: extranodal extension

In our analysis of OS using O’Brien staging, compared with N0P1, only N2P2/N2P3 exerted a tending significant impact on OS (*p* = 0.090, HR: 4.29, 95% CI: 0.80-23.06). The other stages did not significantly alter the risk of death. Other independent factors included age > 65 years, immunosuppression, T3/4 stage, positive margins, and ENE of the cervical LN (Table 3). The C-index was 0.618 (95% CI: 0.608–0.628). The AIC and BIC were 505 and 567, respectively.

### New LN assessment

**Fig. 1:**
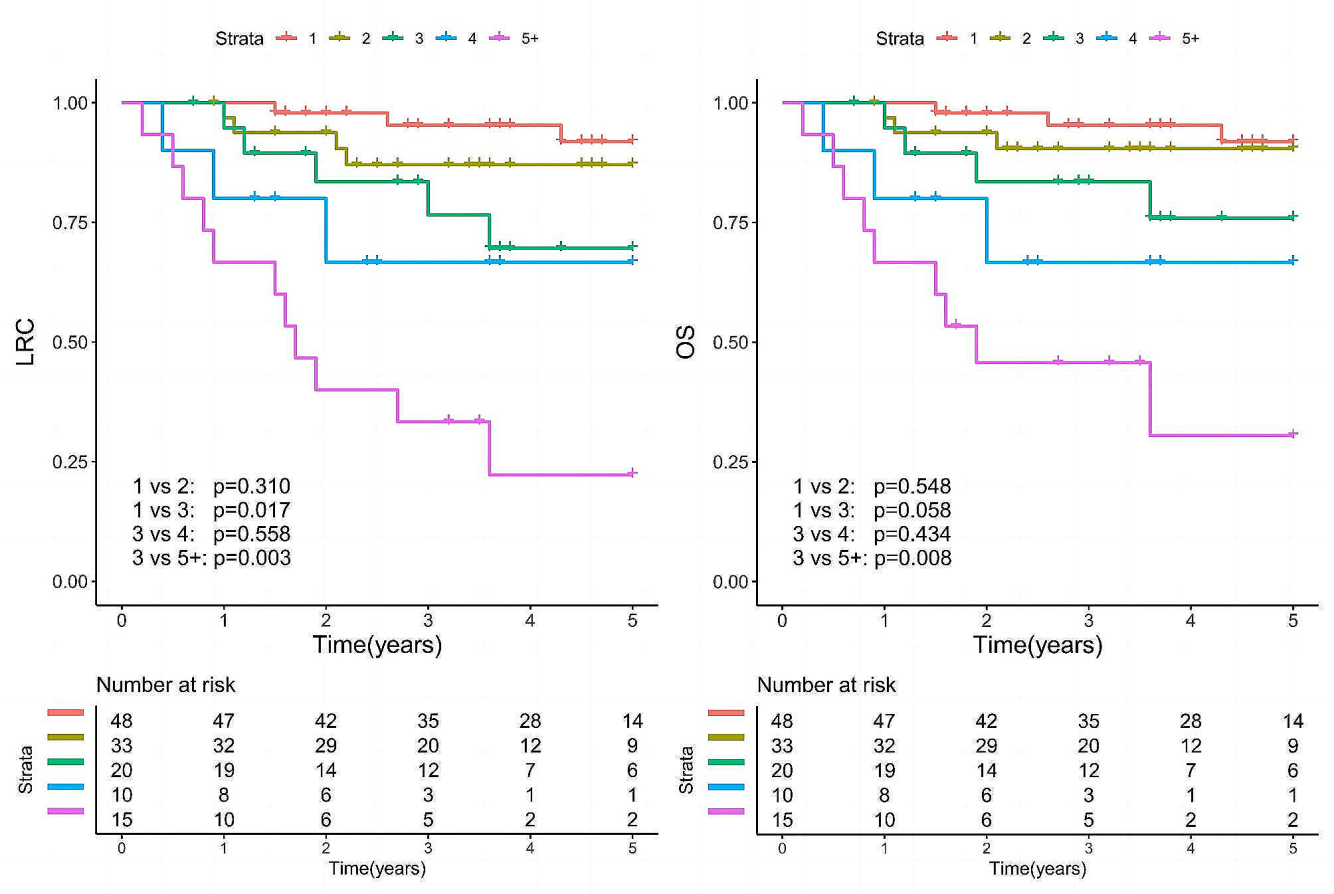
Locoregional control (LRC) and overall survival (OS) in patients with different numbers of metastatic lymph nodes

**Fig. 2:**
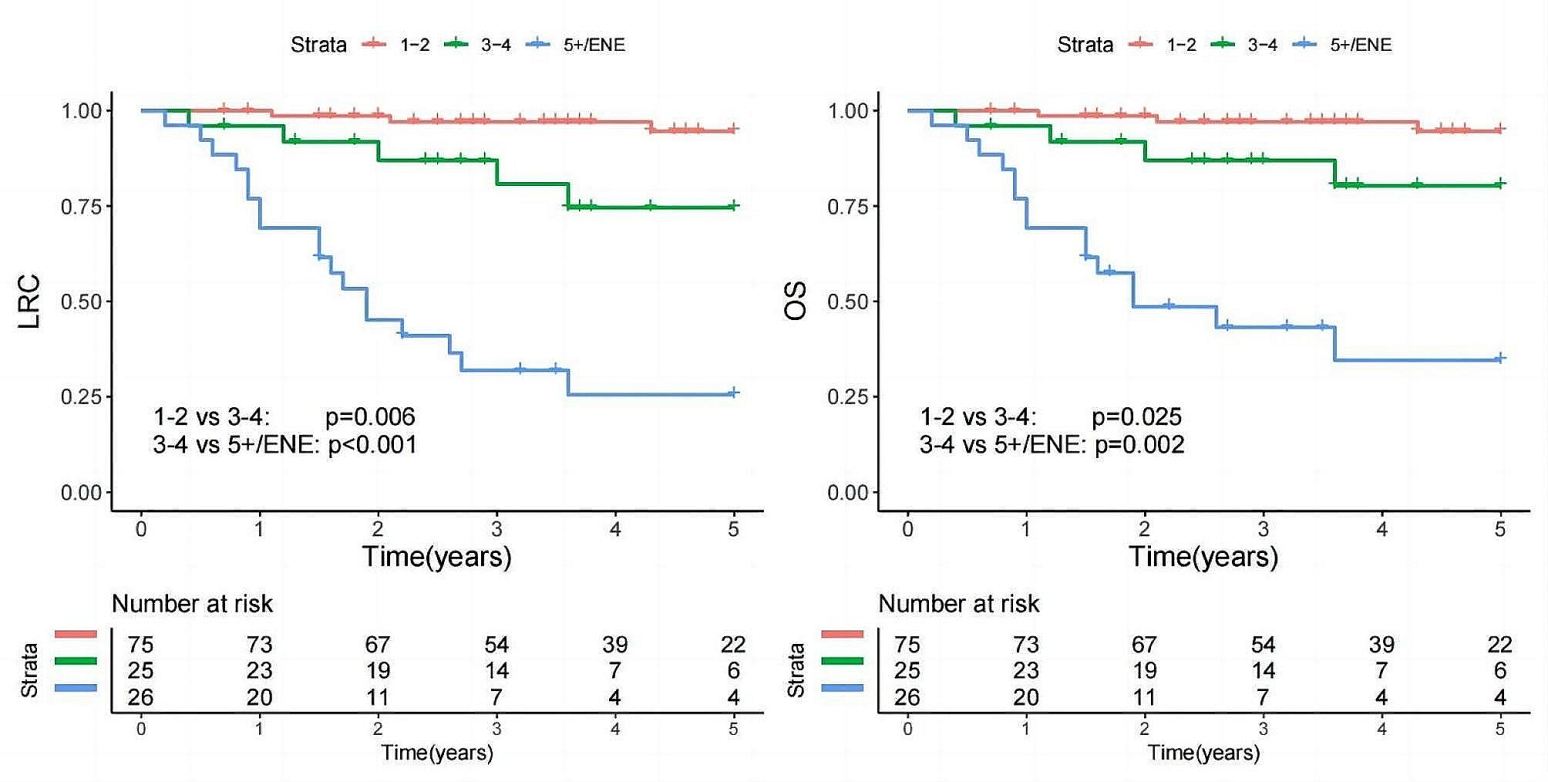
Locoregional control (LRC) and overall survival (OS) in patients stratified by our proposed lymph node stage

The total distribution of metastatic LNs among the patients was as follows: one positive LN in 48 patients, two in 33 patients, three in 20 patients, four in 10 patients, and five or more in 15 patients. The impact of LN metastasis on LRC and OS was assessed by categorizing patients based on the number of positive LNs (1 vs. 2 vs. 3 vs. 4 vs. 5+; Fig. 1). An exploratory analysis revealed that classifying the number of LN metastases as 1–2 vs. 3–4 vs. 5 + yielded the optimal performance. As a result, a new LN staging system was proposed: N1 would denote the presence of 1–2 metastatic LNs, N2 would signify 3–4 positive LNs, and N3 would correspond to 5 or more positive LNs or extranodal extension (ENE) of the cervical LN (Fig. 2).

In our multivariable analysis of LRC utilizing the proposed new staging system, a comparison with N1 revealed that both N2 and N3 exhibited significant disparities, with HRs of 6.80 (95% CI: 1.55–29.95) and 20.12 (95% CI: 5.16–78.51), respectively. Independent prognostic factors included immunosuppression, T3/4 stage, and positive margins (Table 4). The C-index was 0.688 (95% CI: 0.684–0.692), with corresponding AIC and BIC values of 410 and 422, respectively.

**Table 4:** Multivariable analysis of locoregional control (LRC) and overall survival (OS) based on proposed lymph node assessment method

Variable	LRC		OS	
	*p*	HR [95%CI]	*p*	HR [95%CI]
Age (> 65/≤65)			0.047	2.90 [1.01–8.30]
Immunosuppression	0.014	2.49 [1.86–8.28]	0.022	3.60 [1.20−10.82]
Tumor stage (T3−4 vs. T1−2)	0.006	2.30 [1.97–5.46]	0.017	3.96 [1.79–8.86]
Positive margin	< 0.001	29.39 [7.67–112.60]	< 0.001	22.50 [5.79–87.46]
Proposed stage				
N1				
N2	0.011	6.80 [1.55–29.95]	0.020	6.43 [1.35–30.67]
N3	< 0.001	20.12 [5.16–78.50]	< 0.001	13.89 [3.26–59.09]
Radiotherapy	0.099	0.89 [0.77–1.18]	0.087	0.94 [0.86–1.32]

In our multivariable analysis of OS using the new staging system, both N2 (HR: 6.43, 95% CI: 1.35–30.67) and N3 (HR: 13.89, 95% CI: 3.26–59.09) predicted worse prognoses than N1. Other significant variables included age > 65 years, the presence of some form of immunosuppression, T3/4 stage, and positive margins (Table 4). The C-index was 0.675 (95% CI: 0.670–680). The AIC and BIC was 438 and 448, respectively.

## Discussion

Our study’s key discovery highlighted the substantial impact of parotid LNs on the prognosis of HNcSCC, our proposed LN assessment, which considered the number of metastatic LNs and ENE, demonstrated survival stratification similar with the AJCC N or O’Brien staging systems. This innovative approach may offer considerable advantages in facilitating the prompt administration of appropriate treatments to high-risk patients facing recurrence or mortality.

The impact of parotid LNs on HNcSCC has been extensively discussed, given its role as a primary lymphatic drainage site for most HNcSCCs. Both Creighton et al. [4] and Dür et al. [9] concurred that metastasis to parotid LNs was correlated with an elevated likelihood of positive cervical disease. They noted that the sensitivity and negative predictive value of parotid metastasis in diagnosing neck metastasis could be as high as 100%. However, the specific effect of parotid LN involvement on survival remained to be fully elucidated. Sood et al. [10] highlighted in a study of 94 HNcSCC patients that parotid metastasis had a notable prognostic influence, with the impact being influenced by the size of the involved LN. Each 1-cm increase in LN size was associated with a 27% elevated risk of recurrence or mortality, independent of the number of metastatic LNs present. In another study, Myers et al. [11] demonstrated that patients with positive cervical or parotid disease exhibited significantly reduced survival compared to those without metastasis, with a HR of 2.30 (95% CI: 1.27–4.14). Our findings aligned with these reports and present, for possibly the first time, the significance of periparotid LN involvement. We observed that metastasis to this LN predicted inferior LRC and OS. Notably, periparotid LN biopsies can be easily obtained during surgery, and their frozen sections could inform the management of neck-related malignancies [12] and provide guidance for aggressive therapeutic interventions. Previous literature and our analysis collectively emphasized the substantial impact of parotid LN status on survival outcomes, underscoring the importance of incorporating this factor in LN staging considerations [13].

ENE represents another crucial factor that warrants consideration, often serving as an indicator for adjuvant chemotherapy and being encompassed within the AJCC N staging system [14]. In our patient cohort, ENE was observed in 25.0% of intraparotid LNs and 33.3% of periparotid LNs; however, it did not exhibit a significant impact on LRC or OS. In contrast, ENE was present in 34.5% of cervical LNs and demonstrated a substantial adverse effect on prognosis. This disparity could potentially be elucidated by two main factors. Firstly, parotid LNs are typically smaller in size compared to cervical LNs, which may allow even small loci to escape the LN capsule more easily. Secondly, the capsule of parotid LNs is thinner than that of cervical LNs [15], suggesting that the presence of ENE within the parotid LN may be more reflective of anatomical characteristics rather than biological implications. Although previous studies have reported ENE frequencies exceeding 50% in this context [16], Grammatica et al. [17] examined 89 HNcSCC patients and noted ENE rates of 80.0% in cervical LNs and 85.0% in parotid LNs, yet found minimal impact on OS. Similarly, in a study involving 268 HNcSCC patients, ENE was detected in 35.0% of cases but did not demonstrate associations with OS or disease-specific survival [18]. Conversely, our analysis revealed that ENE in cervical LNs, rather than parotid LNs, was indicative of poorer prognosis [7, 13]. These discordant findings may stem from variations in study methodologies, as prior investigations often analyzed ENE as a singular variable encompassing both cervical and other LNs.

An optimal LN staging system should ideally be user-friendly for clinical implementation while demonstrating precise survival stratification. Various LN staging systems exist for metastatic HNcSCC, with the AJCC N stage being the most commonly utilized. This system comprises six levels, with N2c denoting contralateral LN metastasis. Notably, this occurrence is exceptionally rare in HNcSCC cases, representing only 0.7% of 1,128 patients [8]. The prognostic efficacy of this staging system has been scrutinized across numerous studies. For instance, Liu et al. [19] assessed the survival stratification accuracy between the 7th and 8th editions of the AJCC N staging in 382 cases of metastatic HNcSCC. In the 7th edition, N3 status was linked to diminished OS; however, in the 8th edition, no significant differences in OS were observed among N1, N2, and N3 classifications or between stages III and IV. Correspondingly, Luk et al. [20] presented converging perspectives, indicating that the 8th AJCC N stage effectively served as a prognostic indicator.

The O’Brien and N1S3 systems represent two additional methods that have gained significant clinical acceptance [5, 7], with both frameworks incorporating considerations for parotid LNs. The O’Brien stage, characterized by its complexity with nine subgroups, was developed based on clinical parameters but demonstrated suboptimal prognostic stratification when integrated with pathological data. In a previous study involving 89 patients [17], with the majority categorized as N0P2 (21.3%), followed by N0P1 (15.7%), N2P0 (15.7%), and N0P3 (13.5%), all other stages exhibited frequencies of less than 10% in the multivariable analysis. Relative to N0P1, only N2P0, N2P1, and N2P3 revealed reduced impacts on OS, while the remaining stages showed comparable death risks. In line with these observations, our present investigation revealed that, in comparison to N0P1, notable impacts on LRC only tended to manifest with N0P3/N1P1 and N2P2/N2P3, while N2P2/N2P3 appeared to have a bearing on OS. In contrast, the N1S3 staging system factors in both the size and quantity of metastatic LNs, with 5-year OS rates of 78% for stage I, 69% for stage II, and 41% for stage III in the training cohort; however, this distinction was not reproducible in the validation cohort [7]. Notably, ENE was not accounted for in the N1S3 system despite its recognized impact on survival outcomes. Our investigation revealed that the N1S3 staging failed to predict either LRC or OS in univariate analyses.

Based on the number of metastatic LNs and ENE of the cervical LN, a three-category LN stage was proposed (1–2 vs. 3–4 vs. 5 + and/or ENE). This innovative staging approach not only enhanced risk stratification for LRC and OS but also demonstrated similar performance compared to the AJCC N classification and O’Brien stage, as evidenced by improved AIC, BIC, and C-index values. Furthermore, this system achieved a balanced distribution within each group, with representation percentages of 59.5%, 19.8%, and 20.6% in the cohort, respectively. While Ebrahimi et al. [8] also introduced a comparable cutoff value for LN metastasis burden, they overlooked the consideration of ENE as an independent prognostic factor. Our newly proposed staging system represents a substantial advancement, offering enhanced simplicity in application and heightened accuracy levels, thereby signifying a notable improvement in risk stratification for HNcSCC patients.

However, it is essential to recognize the limitations of the present study. Primarily, the retrospective nature of the study introduced selection bias. Secondly, the small and heterogeneous patient cohort restricted the statistical robustness of the findings. Thirdly, additional validation of the newly proposed staging system in larger, preferably prospective, multi-center studies is imperative to affirm its efficacy and generalizability.

In conclusion, the involvement of parotid LNs significantly impacts the prognosis of metastatic HNcSCC. Our suggested LN evaluation approach (1–2 vs. 3–4 vs. 5 + or ENE) could demonstrate similar survival stratification efficacy to the AJCC N and O’Brien systems.

## Data Availability

All data generated or analyzed during this study are included in this published article. And the primary data could be achieved from the corresponding author.
